# *CDC73* c.1155-3A>G is a pathogenic variant that causes aberrant splicing, disrupted parafibromin expression, and hyperparathyroidism-jaw tumor syndrome

**DOI:** 10.1093/jbmrpl/ziae149

**Published:** 2024-11-19

**Authors:** Leor Needleman, Nicolette Chun, Sathvika Sitaraman, Marilyn Tan, Deborah E Sellmeyer, Electron Kebebew, Justin P Annes

**Affiliations:** Department of Medicine, Division of Endocrinology, Stanford University, Stanford, CA 94305, United States; Department of Genetics, Stanford University, Stanford CA 94305, United States; Stanford Cancer Institute, Stanford University, Stanford, CA 94305, United States; Department of Medicine, Division of Endocrinology, Stanford University, Stanford, CA 94305, United States; Department of Medicine, Division of Endocrinology, Stanford University, Stanford, CA 94305, United States; Department of Medicine, Division of Endocrinology, Stanford University, Stanford, CA 94305, United States; Stanford Cancer Institute, Stanford University, Stanford, CA 94305, United States; Department of Surgery, Stanford University, Stanford CA 94305, United States; Department of Medicine, Division of Endocrinology, Stanford University, Stanford, CA 94305, United States; Stanford Cancer Institute, Stanford University, Stanford, CA 94305, United States

**Keywords:** HPT-JT, primary hyperparathyroidism, *Cdc73*, aberrant splicing, RNA sequencing

## Abstract

Germline and somatic pathogenic variants in the *CDC73* gene, encoding the nuclear protein parafibromin, increase the risk for parathyroid carcinoma and cause hereditary primary hyperparathyroidism (PHPT) syndromes known as familial isolated hyperparathyroidism (FIHP) and hyperparathyroidism-jaw tumor syndrome (HPT-JT). The identification of pathogenic germline variants in PHPT-susceptibility genes can influence surgical planning for parathyroidectomy, guide screening for potential syndromic manifestations, and identify/exonerate at-risk family members. Numerous types of pathogenic germline variants have been described for *CDC73*-related conditions, including deletion, truncating, missense, and splice site mutations. Here, we report identification of a non-coding germline *CDC73* variant (*CDC73* c.1155-3A > G), previously categorized as a variant of uncertain significance (VUS), in a family with HPT-JT. This variant, found in two family members with PHPT, altered *CDC73* splicing in peripheral blood cells and disrupted parafibromin immunostaining in associated parathyroid adenomas, strongly evidencing its pathogenicity. Sestamibi scintigraphy yielded nondiagnostic localization results for both patients’ parathyroid adenomas, consistent with prior studies suggesting lower sensitivity for small or cystic lesions. Our findings demonstrate key aspects of *CDC73*-related disorders, highlight the diagnostic value of RNA testing, and exemplify the importance of obtaining a thorough, three-generational family history.

## Graphical Abstract

**Graphical Abstract:**
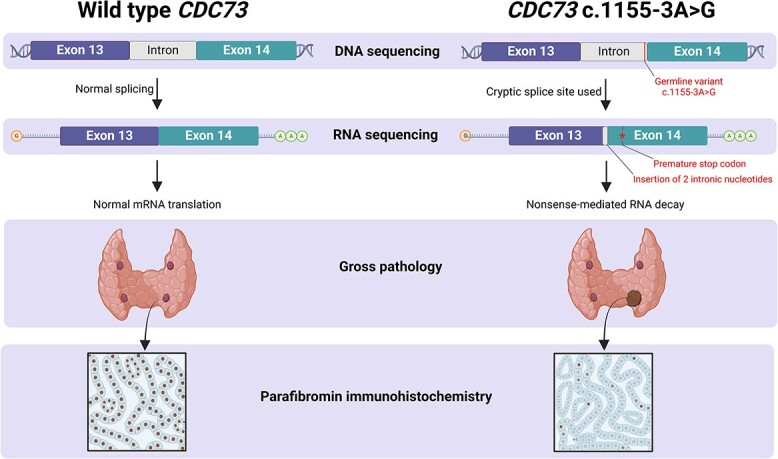

## Introduction

Primary hyperparathyroidism (PHPT) is a disorder of dysregulated parathyroid hormone (PTH) secretion characterized by hypercalcemia with inappropriately elevated PTH levels and increased risk for skeletal and renal disease.^1^ Most cases of PHPT are caused by a single parathyroid adenoma, while a minor proportion result from polyglandular parathyroid hyperplasia. Multiple parathyroid adenomas and parathyroid carcinoma are relatively rare causes of PHPT.

Most patients develop PHPT sporadically as an isolated disorder. However, it is estimated that approximately 10% of PHPT results from a germline mutation in one of 11 susceptibility genes.^2^^,^^3^ Hereditary PHPT can manifest as an isolated disease (familial isolated hyperparathyroidism, FIHP) or as a feature of multiple endocrine neoplasia syndromes (MEN) and hyperparathyroidism-jaw tumor syndrome (HPT-JT). HPT-JT is caused by heterozygous mutations in the *CDC73* gene (previously named HRPT2), which encodes an evolutionarily conserved and ubiquitously expressed nuclear protein called parafibromin.^4^ Somatic and germline *CDC73* variants are identified in a high proportion of patients with parathyroid carcinoma, with some estimates suggesting *CDC73* mutation in two-thirds of all parathyroid carcinoma cases.^5–8^

The absence of parafibromin nuclear staining is useful for diagnosing *CDC73*-related parathyroid tumors.^9^ A tumor suppressor role for parafibromin emerged from examination of *CDC73*-related conditions, whereby germline inactivating mutation and loss of heterozygosity at the *CDC73* locus were identified as genetic drivers of parathyroid neoplasia. In the present study, we report a *CDC73* splice site variant (NM_024529.5:c.1155-3A > G), which has not been previously described in PHPT or HPT-JT. RNA testing demonstrated aberrant *CDC73* splicing in family members harboring the *CDC73* c.1155-3A > G variant and clinical features of HPT-JT. Radiographic findings were consistent with other studies reporting a lower sensitivity for functional parathyroid imaging compared to anatomic imaging for cystic parathyroid adenomas.

## Case 1: proband

### Presentation

An asymptomatic 37-yr-old male was referred for evaluation of hypercalcemia. His medical history included the tetralogy of Fallot, for which he received intracardiac repair during infancy. He had five healthy siblings, although a sister was recently found to have hypercalcemia. His father was diagnosed with PHPT at age 51 and underwent parathyroidectomy (and total thyroidectomy for multinodular goiter) with removal of a 1.8 cm parathyroid adenoma (Table 1). The proband did not have children. His vital signs were normal, and a physical examination was negative for jaw deformities or a palpable neck mass.

**Table 1:** Characteristics of affected family members.

Patient	Genotype	Age at PHPT diagnosis	PHPT complications	Parathyroid surgery	Pathology	Extra-parathyroid tumors^b^	Additional *CDC73*-related conditions
**Proband**	*CDC73* c.1155-3A > G	37	Hypercalcemia, hypercalciuria, osteoporosis	Focused/unilateral parathyroid exploration, left upper parathyroidectomy	Parathyroid adenoma, 1.5 cm, 0.95 g	None	None
**Sister**	*CDC73* c.1155-3A > G	51	Hypercalcemia, hypercalciuria, osteoporosis, nephrolithiasis	Focused/unilateral parathyroid exploration, left lower parathyroidectomy	Cystic parathyroid adenoma, 2.5 cm, 2.8 g	Breast	Renal cyst, uterine cyst^c^
**Father^a^**	Unknown	51	Hypercalcemia, hypercalciuria	Bilateral neck exploration, right upper parathyroidectomy	Parathyroid adenoma, 1.8 cm	Pancreas, multinodular goiter	Unknown

a Complete screening for PHPT complications, germline variants, and nonparathyroid *CDC73*-related conditions unavailable for the father.

b None of the family members were diagnosed with parathyroid tumor metastasis or recurrence.

c Uterine cyst is not represented on the initial spectrum of uterine pathology reported for patients with HPT-JT by Bradley et al.^16^

Abbreviation: PHPT, primary hyperparathyroidism.

### Diagnosis

**Figure 1:**
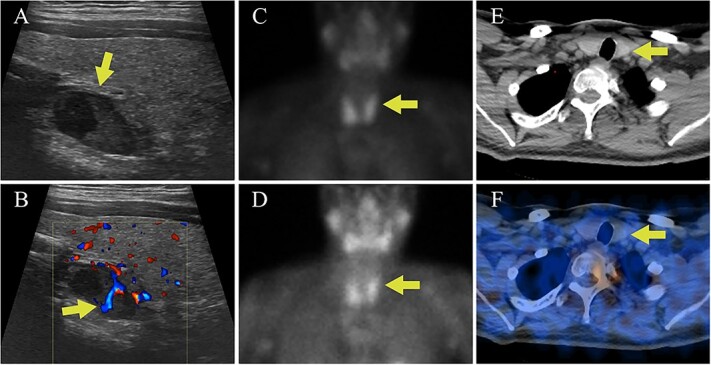
Case I parathyroid localization studies. (A) Thyroid US showed a heterogenous hypoechoic lesion posterior to the left thyroid lobe measuring 0.9 cm deep × 0.9 cm wide × 1.5 cm long (arrow, sagittal view). (B) Internal vascularity within the lesion was evident using doppler imaging (arrow, sagittal view). (C) On technetium-99m sestamibi scintigraphy physiologic uptake or the radiotracer was seen in the thyroid gland on the immediate images (arrow). (D) One hour delayed sestamibi images showed poor washout of radiotracer (arrow) but no areas of focal radiotracer retention. (E, F) No focal areas of uptake were observed on SPECT/CT images in the region correlating with the lesion identified on US (arrows).

The biochemical evaluation was consistent with PTH-dependent hypercalcemia with a total serum calcium of 11.0 mg/dL (2.75 mmol/L, reference 8.6-10.3 mg/dL), albumin 4.6 g/dL (reference 3.6-5.1 g/dL), concurrent PTH 111 pg/mL (reference 14-64 pg/mL), and 25OHD 16 ng/mL (39.94 nmol/L, reference 30-100 ng/mL). Hypercalciuria was also present, with a calcium excretion of 384 mg/24 hr (reference 55-300 mg/24 hr) and a calcium:creatinine clearance ratio of 0.02. After a diagnosis of PHPT was made, DXA showed *Z*-scores of −1.9 at the hip, and −2.2 at the spine and 1/3 forearm. Parathyroid localization with neck ultrasound (US) revealed a 1.5 cm solid, heterogenous, hypoechoic nodule posterior to the left thyroid lobe (Figure 1A and B). However, technetium-99m sestamibi scintigraphy and single photon emission computed tomography/CT (SPECT/CT) did not show any areas of focal uptake (Figure 1C-F).

**Figure 2:**
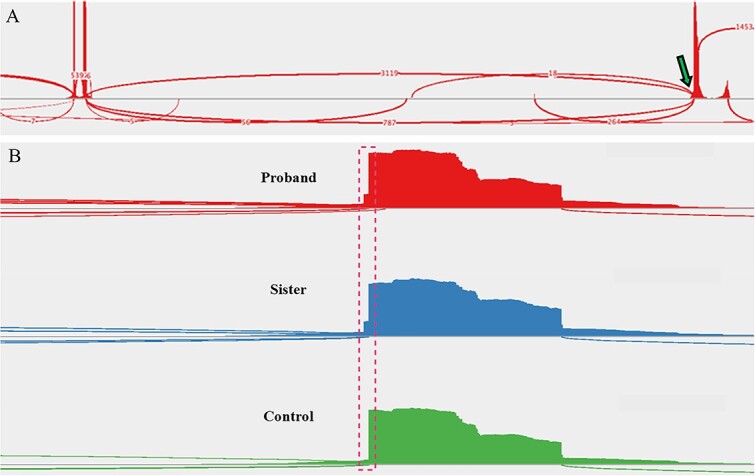
Sashimi plots drawn from RNA-sequencing data. (A) A sample of the proband’s peripheral blood demonstrated the native splice site (arrow, ~80% use) and introduction of a cryptic splice site (~20% use). Read densities at splice junction sites are shown; junction reads are plotted as arcs. (B) A zoomed-in image of the splice junction site reveals the insertion of two intronic nucleotides (dashed box) in the affected family members but not in an unaffected control patient (proband, (top); sister, (middle); control, (bottom)).

The young age of the proband and the presence of hypercalcemia and a parathyroid adenoma in first-degree relatives raised concern for familial PHPT. Genomic DNA was isolated from the proband’s peripheral blood, and targeted regions (within 84 cancer susceptibility genes) were enriched using a hybridization-based protocol and then sequenced (Illumina). The transcript coding sequence and 20 bases of flanking intronic DNA were analyzed. Germline testing identified a heterozygous variant of uncertain significance (VUS) in a non-coding region of the *CDC73* gene (*CDC73* c.1155-3A > G), which had not been reported in association with *CDC73*-related conditions. The c.1155-3A > G sequence substitution occurred at a highly conserved position three nucleotides upstream of exon 14, adjacent to the acceptor splice site sequence. However, because DNA sequencing is insufficient to determine whether the variant induces abnormal splicing, RNA analysis of peripheral blood was obtained (Invitae). From a separate blood sample, RNA was isolated, and complementary DNA for a similar gene panel was synthesized by reverse transcription. The targeted genes were enriched using capture hybridization and then sequenced (Illumina) and aligned to a reference sequence to identify exon junctions, allowing quantitative assessment of splice junction usage. The methods employed revealed the *CDC73* c.1155-3A > G variant activates a cryptic splice site, which leads to the insertion of two intronic nucleotides (r.1154_1155ins1155-2_1155-1, Figure 2). The insertion results in a frameshift at the start of exon 14 and the introduction of a stop codon 20 amino acids downstream, which is predicted to lead to nonsense-mediated RNA decay. Measurement of evolutionary conservation using the UCSC Genome Browser indicates that the nucleotide at the −3 position is conserved (phyloP 2.3967), substantially more so than bases immediately upstream (Figure S1). Hence, RNA analysis indicates that this mutation introduces a cryptic splice site and, based upon evolutionary conservation, may also suppress the efficiency of native splice site utilization.

Screening for additional manifestations of *CDC73*-related disease in the proband was negative, including panoramic jaw X-rays and renal US done to evaluate for jaw and renal tumors, respectively.

### Treatment

**Figure 3:**
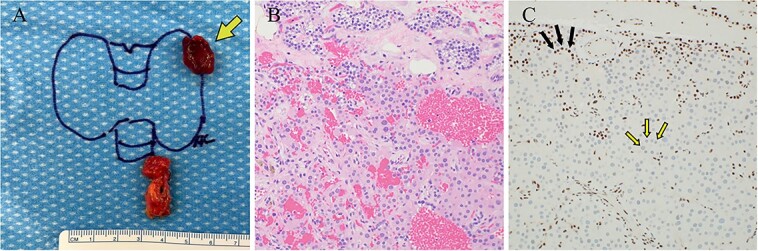
Case 1 left upper parathyroideçtomy pathology. (A) Gross pathology following surgical parathyroid exploration in which the left upper parathyroid gland (arrow) and a portion of the thymus were removed. The excised parathyroid gland measured 1.5 × 1.2 × 0.7 cm and weighed 0.95 g. (B) H&E-stained sections revealed hypercellular parathyroid parenchyma consistent with a parathyroid adenoma. (C) Immunohistochemical staining for parafibromin revealed focally weak to negative nuclear expression in lesional cells (two-toned arrows) with retained staining in surrounding cells (internal positive control, solid arrows).

The patient began supplementation with cholecalciferol 2000 U daily, and the 25OHD level improved to 31 ng/mL (77.38 nmol/L). He underwent left unilateral neck exploration and left upper parathyroidectomy. The baseline pre-excision PTH was 103 pg/mL. At the time of parathyroid gland manipulation, the PTH was 107 pg/mL, and 10 min after excision, the PTH decreased to 37 pg/mL. There were no gross features of parathyroid cancer intraoperatively, and the resected parathyroid gland measured 1.5 cm and weighed 0.95 g (Figure 3A). The patient did not experience any surgical complications and had an uncomplicated recovery. Parathyroid pathology revealed a hypercellular gland consistent with an adenoma, with loss of nuclear parafibromin immunohistochemical staining (Figure 3B and C). One year after surgery, the patient’s serum calcium and PTH levels remained in the normal range (calcium 9.4 mg/dL (2.35 mmol/L), PTH 56 pg/mL). Bone mineral density increased by 5.8% and 4.1% at the spine and hip, respectively, but decreased by 3.6% at the 1/3 forearm. Osteoporosis pharmacotherapy was deferred considering the patient’s relatively young age and absence of fractures. Genetic testing of living first-degree relatives was recommended.

## Case 2: sister of the proband

### Presentation

The proband’s sister with hypercalcemia was evaluated after the proband’s germline *CDC73* variant was determined to have a high probability of being pathogenic. She was 51 yr old and had a history of high-grade left breast ductal carcinoma in situ (DCIS), which was completely resected with mastectomy 6 yr earlier with no subsequent radiation or endocrine therapy. She had two healthy children, and menopause occurred at the age of 45. She developed vaginal bleeding soon after menopause; pelvic US revealed endometrial cysts, although pathology following dilation and curettage only showed inactive endometrium.

### Diagnosis

**Figure 4:**
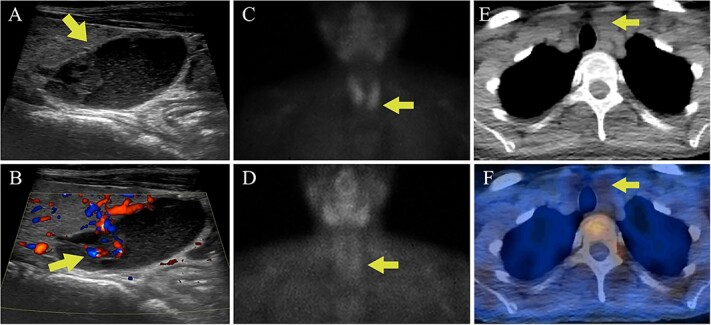
Case 2 parathyroid localization studies. (A) Thyroid US revealed a left inferior thyroid lobe lesion measuring 1.5 cm deep × 1.6 cm wide × 2.0 cm long (arrow, sagittal view). The lesion had a complex cystic and solid appearance with layering internal debris. (B) The solid component of the lesion demonstrated vascularity (arrow, sagittal view with Doppler). (C) Sestamibi scintigraphy showed physiologic radiotracer uptake of the thyroid gland on the immediate images (arrow). (D) One hour delayed sestamibi images showed radiotracer washout without areas of focal sestamibi retention (arrow). (E) The lesion observed on thyroid US correlated with a hypoattenuating lesion posterior to the left thyroid lobe on the CT scan that was done for (arrow). (F) The hypoattenuating lesion did not have increased radiotracer uptake on the fused SPECT/CT image (arrow).

Serum calcium levels available from the time of breast cancer diagnosis ranged between 10.8 and 11.1 mg/dL (2.70 and 2.77 mmol/L). She denied symptoms of hypercalcemia or a history of nephrolithiasis or adult fractures. She was also diagnosed with PHPT after the biochemical assessment showed a serum calcium of 11.1 mg/dL (2.77 mmol/L), albumin 4.6 g/dL, PTH 176 pg/mL, and 25OHD 18 ng/mL (44.93 nmol/L). Urine calcium excretion was 294 mg/24 hr with a calcium:creatinine clearance ratio of 0.02, and renal US showed that her left kidney had both a 4 mm non-obstructing stone and an inferior pole cyst. DXA revealed a lowest T-score of −3.4 at the spine (hip −2.1, 1/3 forearm −2.0). Neck US showed a 2 cm complex cystic and solid structure posterior and inferior to the left thyroid lobe (Figure 4A and B). Sestamibi scintigraphy did not show any areas of abnormal radiotracer uptake (Figure 4C-F). Genetic testing (including breast cancer susceptibility genes) confirmed that the familial *CDC73* c.1155-3A > G variant was present. RNA analysis was performed using the same methods that were employed for the proband and similarly demonstrated aberrant splicing (Figure 2B).

### Treatment

**Figure 5:**
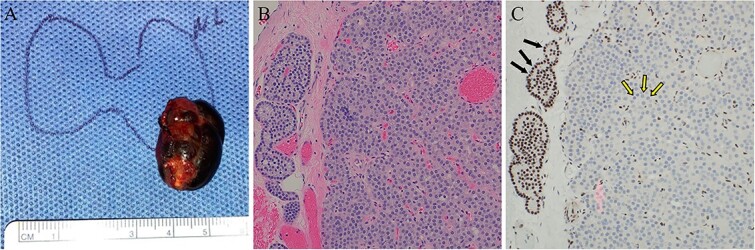
Case 2 left lower parathyroidectomy pathology. (A) The specimen measured 2.5 × 1.5 × 1.2 cm, weighed 2.8 g, and contained a cystic structure consisting of dark brown fluid. (B) H&E-stained sections displayed hypercellular parathyroid parathyroid with associated areas of fibrosis and cyst formation. No significant cytologic atypia, mitotic activity, or angioinvasion was identified. (C) Immunohistochemical staining for parafibromin revealed negative nuclear expression in the lesional cells (two-toned arrows) and retained staining in surrounding cells (internal positive control, solid arrows).

The proband’s sister underwent focused parathyroid exploration targeting the complex cystic structure. Baseline preoperative PTH was 132 pg/mL, and at the time of dissection PTH was 124 pg/mL. The left lower parathyroid gland was excised, measuring 2.5 cm with cystic degeneration (Figure 5A). At 10 min following removal of the parathyroid gland, the PTH was 29 pg/mL. Intraoperatively, there were no gross locally invasive features or lymphadenopathy. She recovered well from surgery without any complications. H&E staining showed a hypercellular parathyroid gland with cystic change, without malignant features (Figure 5B). Immunohistochemistry showed loss of nuclear parafibromin staining (Figure 5C). One year after surgery, the patient maintained normal calcium and PTH levels (calcium 9.8 mg/dL (2.45 mmol/L), PTH 42 pg/mL). Repeat DXA showed an increase in bone mineral density of 5.7% at the spine, 8.9% at the hip, and 3.7% at the 1/3 forearm. Osteoporosis pharmacotherapy was deferred to monitor for further improvement in bone density, as in the case of the proband. The ensuing screening program for both siblings involved biochemical surveillance for recurrence of PHPT (annual calcium and PTH) and imaging every 5 yr with jaw X-ray and renal US. The proband’s sister also required continued screening for uterine abnormalities with pelvic US every 5 yr, and genetic testing was recommended for her children.

## Discussion

The diagnostic findings presented in this study illuminate several important aspects of hereditary PHPT. Obtaining a careful family history is essential for recognizing hereditary disease. While there is insufficient evidence for an appropriate age cutoff for genetic testing in PHPT, age <25-45 yr is variably suggested in guidelines.^10–13^ Additional indications for genetic testing in PHPT include multiglandular disease, parathyroid carcinoma, atypical parathyroid adenoma, or a relative with hypercalcemia or features of a hereditary PHPT syndrome. A germline mutation in 1 of 11 susceptibility genes, including *CDC73*, is present in about 10% of individuals with PHPT.^2^^,^^3^ Hereditary conditions associated with loss-of-function mutations in *CDC73* include FIHP and HPT-JT. A retrospective cohort study of PHPT patients who underwent germline *CDC73* analysis reported an estimated penetrance of 65% at age 50 for one or more *CDC73*-related disorders.^14^ This, and numerous other studies, have shown that PHPT has the highest penetrance among the *CDC73*-related disorders. Although HPT-JT patients may also develop ossifying jaw fibromas and renal abnormalities (kidney cysts, hamartomas, Wilms tumor, and clear cell carcinoma), the penetrance of jaw or renal involvement is significantly lower than for parathyroid tumors.^15^ In addition, a high proportion of women with *CDC73* variants were found to have various types of uterine abnormalities (adenosarcoma, adenofibroma, leiomyoma, adenomyosis, and endometrial hyperplasia).^16^

The product of the *CDC73* gene is the nuclear protein parafibromin, which is known to have important roles in transcription elongation and RNA processing via interactions with the Paf1 complex and RNA polymerase II.^17^^,^^18^ In addition, parafibromin inhibits cell cycle progression.^18^ Parafibromin directly interacts with p53 mRNA and β-catenin, suggesting alterations in p53 expression and Wnt signaling as potential tumorigenic mechanisms.^19^^,^^20^ The *CDC73* gene is comprised of 17 exons, encoding the 531 amino acid parafibromin protein. In a database of human *CDC73* variants, germline and somatic mutations were enriched in exons 1, 2, and 7.^21^ Among these variants, 79% were frameshift or nonsense, 13% were missense, and 6% involved splice sites. *CDC73* splice site variants have been reported in association with FIHP and HPT-JT.^22–25^ At present, ClinVar, a public archive of human variants, includes 22 germline *CDC73* variants, which are categorized as pathogenic or likely pathogenic and involve splice sites (Table 2). Limited genotype–phenotype correlations for *CDC73* and structural data for parafibromin have been suggested. Mutations that involve residues 136-139 result in loss of nuclear localization, suggesting these amino acids comprise a nuclear localization signal (NLS).^26^ An evolutionarily conserved domain consisting of amino acid residues 218-263 binds to β-catenin.^19^ Mutations expected to have a significant impact on parafibromin function or expression were shown to increase risk for parathyroid carcinoma and jaw disease.^8^ While the mechanism is not fully clear, more damaging mutations may disrupt the C-terminus of parafibromin, which is likely necessary for Paf1 complex recruitment and transcription elongation.^27^

**Table 2:** ClinVar germline pathogenic/likely pathogenic *CDC73* splice site variants.

Variants reported in patients with *CDC73*-related disease		
Genotype	Phenotype	Reference
**c.131+1G>A**	FIHP	Cetani et al.^22^ Bradley et al.^23^
**c.132-2A>G**	FIHP	No citation exists
**c.237+1G>C**	FIHP	Villablanca et al.^24^
**c.238-2A>T**	Undisclosed by submitter	No citation exists
**c.238-1G>A**	HPT-JT	Moon et al.^25^
**c.307+1G>A**	FIHP	Kong et al.^46^
**c.423+1G>A**	HPT-JT	Li et al.^8^
**c.1155-3A>G**	HPT-JT	Present study
**Variants not yet observed in patients with *CDC73*-related disease**		
**Genotype**		
**c.131+1del**		
**c.131+1G>T**		
**c.132-2A>C**		
**c.237_237+1insA**		
**c.237+1G>A**		
**c.237+1G>T**		
**c.238-1G>T**		
**c.513-1G>A**		
**c.729+1G>T**		
**c.1030+1G>A**		
**c.1030+2T>C**		
**c.1067-2_1069del**		
**c.1067-2A>G**		
**c.1316+1del**		

Abbreviations: FIHP, familial isolated hyperparathyroidism; HPT-JT, hyperparathyroidism-jaw tumor.

Parafibromin expression has been shown to be inversely correlated with tumor size and pathological stage in breast, gastric, and laryngeal squamous cell carcinoma.^28–30^ However, whether parafibromin has a tumor suppressor role in tissues other than the parathyroid glands, jaw bones, kidneys, or uterus requires further investigation. In the present study, the proband’s sister was diagnosed with a relatively large, high-grade DCIS tumor at the age of 45. Genetic testing that identified the germline *CDC73* variant included a breast cancer panel but did not identify any other variants. However, parafibromin staining, loss of heterozygosity of the WT *CDC73* allele, and somatic *CDC73* variants were not examined in the DCIS tumor.

The identification of pathogenic germline *CDC73* variants can guide surgical planning for parathyroidectomy, appropriate utilization of screening programs, and genetic testing of family members at risk. Screening for *CDC73*-related disorders has high clinical value because of the risk for parathyroid carcinoma, tumors of the kidney and uterus that may also be malignant, and the potential for locally aggressive jaw tumors. If a pathogenic variant is identified at a young age, screening is recommended to begin during childhood.^31^ Guidelines recommend checking serum calcium and PTH levels annually and jaw X-ray and renal US every 5 yr.^32^ Women of reproductive age should have an annual pelvic exam and pelvic US every 5 yr.

In the present study, the germline variant c.1155-3A > G located within intron 13 of the *CDC73* gene was identified in two family members with PHPT due to solitary parathyroid adenomas. Their father did not complete genetic testing but also had PHPT due to a single parathyroid adenoma. The *CDC73* c.1155-3A > G sequence change (located on chromosome 1 at position 193 232 990 in the GRCh38.p14 assembly) has not been reported in the literature in individuals affected with *CDC73*-related conditions. This variant first appeared in ClinVar (Variation ID: 403885) in 2017 and was initially classified as VUS by Invitae (despite in silico analyses predicting a deleterious effect on splicing) because at the time the variant had not been observed in individuals with *CDC73*-related disease. Since then, two additional clinical laboratories have submitted this variant to ClinVar with VUS classification. In 2022, the variant was identified in the proband of the present study using targeted sequencing (Invitae) of 84 genes associated with cancer predisposition conditions.

The allele frequency of the c.1155-3A > G variant is 6.202 × 10^−7^ for the overall population and 8.485 × 10^−7^ for the non-Finnish European population (the population with the highest allele frequency), consistent with a low prevalence in population databases (gnomAD v4.1.0).^33^ gnomAD also provides a gene constraint score (referred to as observed/expected or “oe” score) based on mutational modeling and depicts a measure of how tolerant a transcript is to variations. The oe score of the surrounding 1 kb region for the c.1155-3A > G variant is 0.898 with a *Z* score of 1.421 (*Z* score range −10 to 10; *Z* score > 2.18 represents the top 10% of constrained non-coding regions), reflecting relatively low genomic constraint. Nevertheless, the c.1155-3A nucleotide is evolutionarily conserved and, when mutated, disrupts *CDC73* expression. In silico splicing variant interpretation tools (SpliceAI 0.990, Pangolin 0.870) predict a high probability that the c.1155-3A > G variant affects splicing, consistent with the peripheral blood cell RNA analysis. Additionally, immunohistochemistry demonstrated pathogenic loss of nuclear parafibromin immunostaining.

The family reported here provided integral evidence supporting the reclassification of the *CDC73* c.1155-3A > G variant by Invitae as likely pathogenic. This variant satisfies criteria for “likely pathogenic” set forth by the American College of Medical Genetics and Genomics (ACMG) guidelines for the interpretation of sequence variants.^34^ Specifically, functional data demonstrating a variant’s impact on RNA transcription or stability represents strong evidence of pathogenicity. RNA analysis in conjunction with the computational splicing data and cosegregation of the variant with family members affected by *CDC73*-related disease provided sufficient proof to appropriately change the classification of the *CDC73* c.1155-3A > G variant from VUS to likely pathogenic using the rules for combining criteria to classify variants in the ACMG guidelines.

Distinguishing features of parathyroid tumors reported in association with HPT-JT include asynchronous multiglandular involvement, increased risk of malignancy, and a tendency to develop cystic changes. The authors of an early pathologic study of HPT-JT families initially termed the syndrome “familial cystic parathyroid adenomatosis.”^35^ HPT-JT, rather than FIHP, is the most suitable clinical characterization for this family given the presence of a cystic parathyroid adenoma and renal and uterine cysts in the sister of the proband. The initial description of uterine abnormalities in patients with HPT-JT does not include uterine cysts, although these may be considered a potential feature of uterine involvement. Moreover, the *CDC73*-related conditions may exist on a spectrum of potential disease manifestations rather than as distinct clinical entities.

Accurate preoperative localization is necessary for parathyroidectomy surgical planning. In patients with HPT-JT, anatomic imaging (US, magnetic resonance imaging (MRI), or four-dimensional CT (4D-CT)) is important as it not only assists with localizing parathyroid tumors but can also provide information about the likelihood of malignancy by assessing for local invasion. This allows surgeons to give better preoperative counseling about the risk of parathyroid carcinoma and the potential need for en bloc resection with the ipsilateral thyroid lobe. Parathyroid imaging guidelines also recommend using functional imaging for preoperative localization with sestamibi scintigraphy.^36^ The visualization of sestamibi radiotracer in parathyroid tissue depends on its accumulation in the mitochondria of parathyroid oxyphil cells.^37^^,^^38^ A meta-analysis of sestamibi SPECT/CT, which leverages hybridization of functional and anatomic data, reported a sensitivity of only 68% for localizing PHPT.^39^ Causes of false negative parathyroid imaging with sestamibi include small gland size, superior position, relatively low oxyphil cell content, use of calcium channel blockers, and coexisting thyroid disease.^40–42^ In addition, several studies have reported low sensitivity of sestamibi scintigraphy for cystic parathyroid adenoma.^43^^,^^44^ A recent meta-analysis of cystic parathyroid adenomas reported a detection rate of 85% (99/117) with neck US, 100% (32/32) with US-guided fine needle aspiration (FNA), and 100% (27/27) for the aggregate of CT, 4D-CT, and MRI.^45^ Sestamibi SPECT detected only 61% (95/157) of cystic parathyroid adenomas. The findings led the authors to propose that a cystic parathyroid adenoma identified on US should be followed by 4D-CT, whereas sestamibi imaging may be considered for solid lesions. The proband of the present study may have had a false-negative sestamibi scan due to small lesion size coupled with superior position, while the sister’s negative imaging may be explained by the presence of a cystic adenoma.

Gathering a thorough family medical history is crucial for the proper management of PHPT. Additional studies are needed to better inform guidelines for genetic testing. Our diagnostic evaluation provided necessary clinical data for the revision of the pathogenicity interpretation of the *CDC73* c.1155-3A > G variant and emphasizes the role of RNA sequencing in the assessment of splice site variants.

## Supplementary Material

Supplementary_Figure_1_ziae149

## Data Availability

No datasets were generated or analyzed during the current study.
