# Supplementary figures and images for: CDC73 c.1155-3A>G is a pathogenic variant that causes aberrant splicing, disrupted parafibromin expression, and hyperparathyroidism-jaw tumor syndrome
Source: JBMR Plus. 2024 Nov 19;9(1):ziae149. doi: 10.1093/jbmrpl/ziae149 (PMC11646312; doi:10.1093/jbmrpl/ziae149)

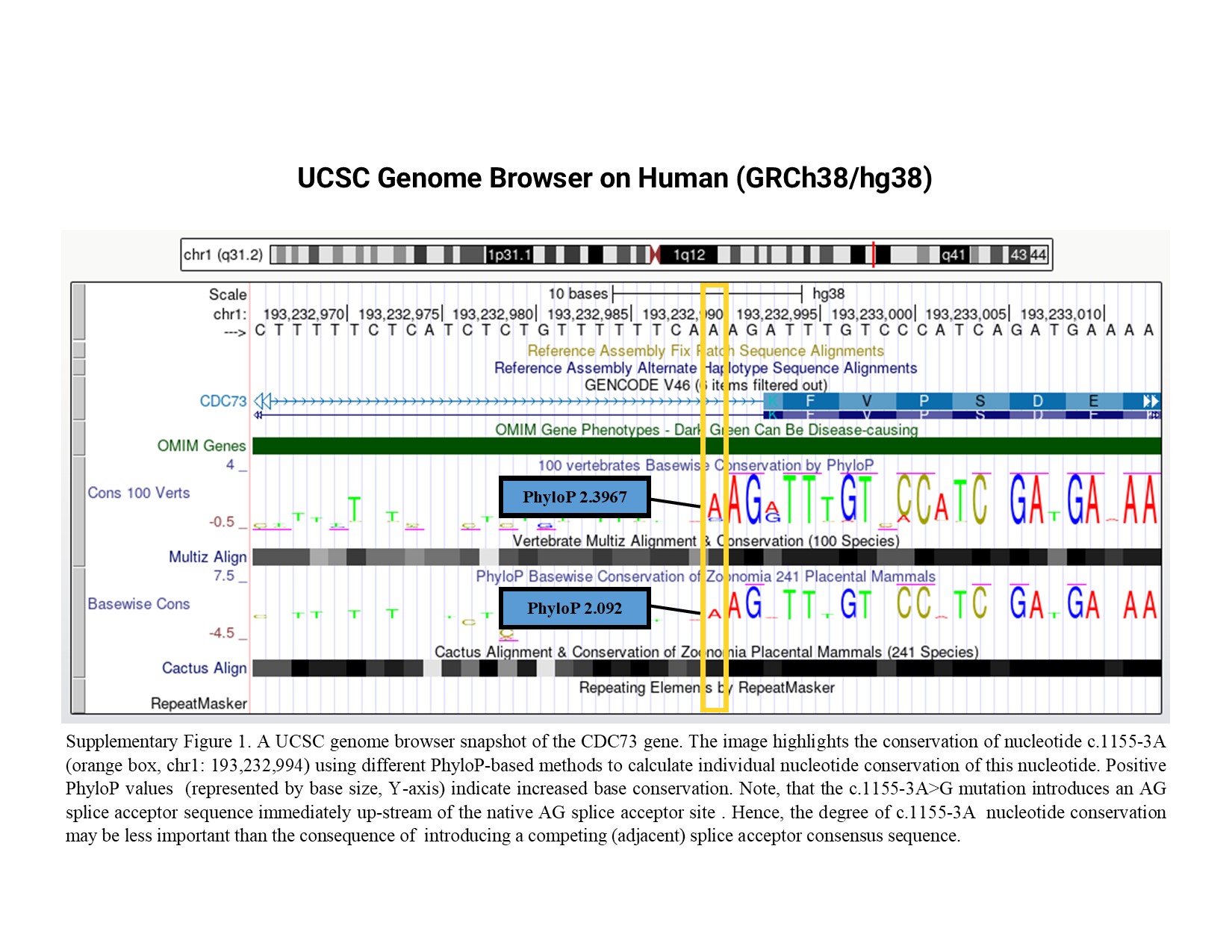

Supplement: Supplementary_Figure_1_ziae149 [file supplementary_figure_1_ziae149.jpeg]
